# Automatic generation of 3D motifs for classification of protein binding sites

**DOI:** 10.1186/1471-2105-8-321

**Published:** 2007-08-30

**Authors:** Jean-Christophe Nebel, Pawel Herzyk, David R Gilbert

**Affiliations:** 1 Faculty of Computing, Information Systems & Mathematics, Kingston University, Kingston-upon-Thames, KT1 2EE, UK; 2 Bioinformatics Research Centre, University of Glasgow, Glasgow, G12 8QQ, UK; 3 The Sir Henry Wellcome Functional Genomics Facility, Institute of Biomedical and Life Sciences, University of Glasgow, Glasgow, G12 8QQ, UK

## Abstract

**Background:**

Since many of the new protein structures delivered by high-throughput processes do not have any known function, there is a need for structure-based prediction of protein function. Protein 3D structures can be clustered according to their fold or secondary structures to produce classes of some functional significance. A recent alternative has been to detect specific 3D motifs which are often associated to active sites. Unfortunately, there are very few known 3D motifs, which are usually the result of a manual process, compared to the number of sequential motifs already known. In this paper, we report a method to automatically generate 3D motifs of protein structure binding sites based on consensus atom positions and evaluate it on a set of adenine based ligands.

**Results:**

Our new approach was validated by generating automatically 3D patterns for the main adenine based ligands, i.e. AMP, ADP and ATP. Out of the 18 detected patterns, only one, the ADP4 pattern, is not associated with well defined structural patterns. Moreover, most of the patterns could be classified as binding site 3D motifs. Literature research revealed that the ADP4 pattern actually corresponds to structural features which show complex evolutionary links between ligases and transferases. Therefore, all of the generated patterns prove to be meaningful. Each pattern was used to query all PDB proteins which bind either purine based or guanine based ligands, in order to evaluate the classification and annotation properties of the pattern. Overall, our 3D patterns matched 31% of proteins with adenine based ligands and 95.5% of them were classified correctly.

**Conclusion:**

A new metric has been introduced allowing the classification of proteins according to the similarity of atomic environment of binding sites, and a methodology has been developed to automatically produce 3D patterns from that classification. A study of proteins binding adenine based ligands showed that these 3D patterns are not only biochemically meaningful, but can be used for protein classification and annotation.

## Background

Structural genomics projects aim at high-throughput delivery of protein structures regardless of the state of their functional annotation. Moreover, roughly half of gene-products delivered by completed genomes of various organisms do not show sequence homology to existing proteins of known function. Therefore, structure-based prediction of protein molecular function is essential. Protein 3D structures can be clustered according to their fold in classes which usually have some functional significance, e.g. SCOP [1], FSSP [2] and CATH [3]. More recently, researchers have investigated the detection of functional 3D patterns associated with active sites or/and atom interactions (see the Methods section for an explanation of the terminology used throughout this paper). These patterns may be based on secondary structures, such as the EF-hand domain and zinc fingers or sets of 3D positions of atoms involved either in H-bonds [4] or ligand binding [5,6]. Moreover, tools have been developed which allow the detection of residue based 3D patterns within a protein structure [5-8] and the comparison of the 3D structure of binding sites with other proteins [9]. Unfortunately, many patterns do not correspond to any specific function and the number of known 3D motifs is rather small compared to the number of sequential motifs captured in databases such as PROSITE [10] and InterPro [11]. So far, 3D motifs have been mainly the result of some manual and experimental process, e.g. the Catalytic Site Atlas [6]. Moreover, patterns are usually constructed on the basis of residues and are represented either by their Cα atoms, atoms interacting with ligands or all their atoms.

Although proteins are composed of amino acids which are very convenient and useful structural units for the analysis of proteins, ultimately chemical interactions happen at the atomic level. The associations of residues in physicochemical groups which are not mutually exclusive implicitly acknowledge the limitation of residue-centred approaches [12]. We propose a new approach where 3D motifs are generated automatically and are based only on consensus atom positions without explicit reference to the residues to which they belong and the direct interactions they may have with ligands. In this work, the ligand binding sites of a protein are compared by superimposing the corresponding ligands. The similarity between ligand environments is then evaluated by calculating the number of atoms of the same type which share equivalent spatial positions. By converting that similarity measure into a normalised metric, a similarity matrix can be generated for a given set of proteins in order to permit clustering of their ligand binding sites. Subsequently, consensus 3D patterns can be produced to represent each of the clusters. Because the clusters can be shown to be associated with specific biochemical functions, protein structures can be compared to these 3D motifs in order to predict their function.

## Results and discussion

### 3D pattern generation for adenine phosphate

**Figure 1:**
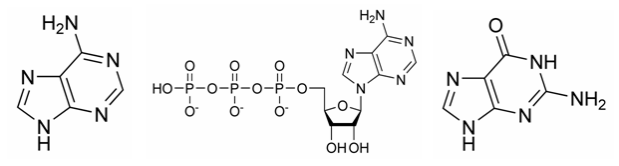
Chemical structures of adenine, ATP and guanine.

We evaluated our method by automatically generating 3D patterns for the main adenine based ligands, i.e. AMP, ADP and ATP. These ligands were selected because they are relatively common, key to many biochemical reactions and contain rigid groups which make their superimposition meaningful. Subsequently, the patterns produced were tested against other adenine based molecules, i.e. ACP and ANP, and guanine based ligands, i.e. GMP, GDP and GTP. Figure 1 shows the main chemical structures involved in the ligands of interest.

For each of the main adenine based ligands, a binding site similarity matrix was generated using ligand-specific training sets (see Methods section). In this work, two atoms of the same chemical type are considered to share a similar position if the distance between them is less than 1.25 Å. This value was chosen because in previous work [13] it was shown to be a good compromise between accuracy and flexibility. The binding sites were then clustered and outliers were removed using a similarity threshold *S*_*T *_= 0.6 (see Method section). The consensus 3D patterns were generated for valid clusters that contain at least 3 binding sites. Table 1 presents statistics associated with the 18 valid clusters identified using three main adenine based ligands AMP, ADP and ATP, including the number of proteins from which they were generated and their core size.

**Table 1:** Initial clusters for each ligand

**Ligand**	**Proteins in PDB50**	**Valid clusters**	**Core cluster sizes**
AMP	75	3	5-4-3
ADP	185	9	10-9-8-7-6-5-5-4-4
ATP	133	6	10-9-4-3-3-3

The 3D patterns corresponding to the above 18 clusters were then compared in an all against all manner. Clusters with highly similar 3D patterns were merged into new 3D patterns, resulting in a reduction of the number of clusters from 18 to 13 [see cluster composition in Additional file 1]. Merging was performed between AMP and ATP, ADP and ATP, but not between AMP and ADP clusters. The similarity threshold associated with each pattern was defined as the lowest similarity score between any pair of binding sites belonging to the cluster represented by that pattern. These values are called the automatic similarity threshold.

### From 3D pattern to 3D motif

Table 2 presents the characteristics of the final 3D patterns in form of cluster sizes, numbers of consensus atoms as well as the consensus information collected from PDBSum [14] for all the proteins contributing to the patterns. The pattern sizes in terms of the number of atoms involved are very different and range from 6 to 71, constituting between 4% and 46% of the average binding site respectively, as there are on average 154 atoms in a binding site [see 3D patterns in Additional files 2, 3, 4, 5, 6, 7, 8, 9, 10, 11, 12, 13, 14].

**Table 2:** Consensus information about each pattern

**Pattern**	**Rep.**	**Ligand**	**Size**	**Atoms**	**Function**	**EC**	**CATH**	**DALI**	**IPR**	**PS**
**ADP0**	1vom	ADP	6	11	Myosin	/	1.10.162.10 1.10.183.10 1.10.465.10 3.30.538.10	1237	001093 001609	
**ADP1**	1q0b	ADP	7	7	Kinesin	/	3.40.850.10	1236	001752	50067 00411
**ADP2**	1b62	ADP	10	8			3.30.565.10	(846, 847, 848)	003594	
**ADP3**	1qf9	ADP	8	23	Transferase	2.7. (2.7.1., 2.7.4.)	3.40.50.300			
**ADP4**	1ehi	ADP	5	18	Ligase (60%) Transferase (40%)		3.30.			
**ADP5**	1njf	ADP	4	17			*1.10.8.60* *3.40.50.300*	1062	003593	
**ADP6**	1oxu	ADP	5	6	Abc transporter	/	3.40.50.300	1069 1071	003593 003439	00211 50893
**AMP0**	1v26	AMP	4	27	Ligase		*2.30.38.10* *3.30.300.30* *3.40.50.980*	736 992	000873	00455
**ATP0**	1y8q	ATP	3	71	Ligase	ND	*3.40.50.70*	*851*	000205 000594	
**AxP0**	2a40	ADP ATP	13	42	Actin (85%) Heat shock (15%)	/	3.30.420.40 3.90.640.10	1175		
**AxP1**	1j1c	ADP ATP	20	45	Kinase	2.7.1.	1.10.510.10 3.30.200.20	593	000719	50011
**AxP2**	1ses	AMP ATP	8	51	Ligase	6.1.1.	3.30.930.10	1116		50862
**AxP3**	1o97	AMP ATP	7	42			3.40.50.620	(973 974 975)		

This table consists of the name of the pattern, its PDB representative (Rep.), names of ligands the pattern was created from, the size of the cluster, the number of atoms in the pattern, the annotated function, the EC (Enzyme Commission) number if relevant [35], the protein fold classifications according to CATH [3] and DALI [2], and finally detected sequence motifs according to InterPro (IPR) [11] and PROSITE (PS) [10]. When consensus values could not be found, but close alternatives were available, values are shown between brackets. Finally, when data was not available through PDBSum, it was inferred when possible using homologues; in this case values are shown in italic. Otherwise when not enough data was available to generate a meaningful consensus value, the code 'ND' is used.

Table 2 demonstrates that all 3D patterns, except ADP4, are associated with well defined structural patterns as described by the CATH and DALI identifiers. This observation validates our similarity metric which is based on the number of common atoms in the neighbourhood of a ligand, as a meaningful metric for protein structure comparison. Since 7 out of the 13 3D patterns also combine consensus function and sequential motifs (ADP0, ADP1, ADP6, AMP0, ATP0, AxP1, AxP2), we can classify them as binding site 3D motifs. Furthermore, although neither ADP2 nor ADP5 are associated with a specific function, each is related to a sequence motif. Therefore they must be linked to sub-functions which are performed by ADP binding and consequently can be classified as 3D motifs. Finally, ADP3 can also be considered as a 3D motif since it has a clear function related to its EC number and it belongs to a unique CATH homologous superfamily. DALI classifies the transferases of ADP3 into two very different folds, which suggests the local similarity of their binding sites is lost in DALI's global structure comparison process.

The case of the 3 remaining 3D patterns (ADP4, AxP0, AxP3) requires further analysis to decide if they can be classified as 3D motifs. Consequently, we decided to check if any of the 13 patterns (Table 2) correspond to structural templates generated from the annotated catalytic sites of CSA [6], see Table 3. Submission of the representatives to the web server provided by CSA returned only three hits which were classified either as probable or highly probable. Since two of them did not target the adenine binding site, only one was relevant to this study. This CSA template matched the binding site of 1ses It corresponds very closely to the AxP2 pattern since out of the 5 residues present in CSA, 3 of them are represented in that pattern.

**Table 3:** 3D patterns detected by other systems

**Pattern**	**Rep.**	**CSA 3D Template**	**SuMo similarity**	**PINTS similarity**
**ADP0**	1vom		**YES**	**YES**
**ADP1**	1q0b			**PROBABLE**
**ADP6**	1oxu			**YES**
**ATP0**	1y8q			**YES**
**AxP0**	2a40			**PROBABLE**
**AxP2**	1ses	**YES**		

This table consists of the patterns which were identified by other systems able to detect 3D motifs (i.e. CSA, SuMo and PINTS).

Since CSA recognised only 1 out of our 13 templates, we investigated if they could potentially be detected by other methods. The adenine binding sites of the representative proteins were submitted to the SuMo server for 3D searches for functional sites [9] and to the PINTS server find 3D local structural patterns [5], which compared them to all ligand binding sites in the PDB [15]. SuMo only detected strong similarities between the binding sites of the core proteins associated with the ADP0 patterns: 5 proteins were ranked very high and only 1 protein was not matched by the site. For PINTS, all core proteins of ADP0 and ATP0 ranked very highly against their cluster representative. The existence of a common pattern was also detected with ADP6 where only 1 protein out of 5 was not highly ranked. Moreover, some evidence of binding site similarity could be collected for ADP1 and AxP0, since respectively 4 out of 7 and 7 out of 13 proteins (all of which are actins) were shown to share similar sites. Results from these three servers confirm that our metric can be used for generating meaningful patterns, most of which are 3D motifs. Moreover, the fact that only 6 of our 13 patterns could potentially have been detected by these systems suggests that our metric captures some new important features from binding sites.

### Structural alignment and complex evolutionary links

Contrary to all the other patterns, ADP4 does not correspond to a class with a common function, structure or sequence pattern. The analysis of the properties of the five proteins which comprise its core reveals that three of them are ligases (1ehi, 1e4e & 1gsa) and two are transferases (1kjq & 1iah). Structurally however, the 1ehi, 1e4e, 1gsa and 1kjq ATP-binding domains are classified by SCOP [1] as belonging to the ATP-grasp fold and the glutathione synthetase ATP-binding domain-like superfamily and share other structure and sequence patterns such as:

CATH 3.30.1490.20, FSSP 1053, 1055 & 1058, IPR011761 and PS50975

The 1iah structure, however, is classified by SCOP as a MHCK/EF2 atypical kinase belonging to the protein kinase-like superfamily and fold; its structure and sequence patterns, namely CATH 3.30.200.20, FSSP 601 & 2482, IPR002111, IPR005821 and IPR004166 do not match the ones for the other ADP4 proteins.

The VAST structure similarity server [16] does not detect any structural neighbour of 1iah within the cluster. Neither does the multiple sequence alignment of these five proteins with either ClustalW [17], MUSCLE [18] or TCoffee [19] show any sequence pattern shared by this cluster. However, their structural alignment constructed using our system highlights the following pattern: [EQ]-X-[ACVY] [MLV], which is completed by two non-polar residues and a conserved Lysine, [FI] [VLI] [K] 36–72 residues upstream. Multiple comparisons with either the MSDfold [20] or CE_MC [21] structure similarity search engines do not detect this pattern.

**Figure 2:**
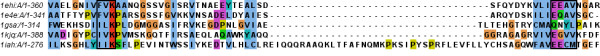
Alignment of the sequences using structural constraints.

**Figure 3:**
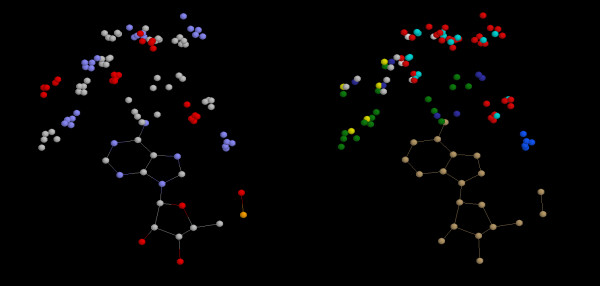
**Common pattern associated to the ADP4 pattern**. Superimposition of atoms from 1ehi, 1e4e, 1gsa, 1kjq & 1iah used for the generation of the pattern. Wireframe shows consensus atoms belonging to the adenine-based ligand. a) CPK colour scheme is used. b) Amino acid/Shapely colour scheme is used.

**Figure 4:**
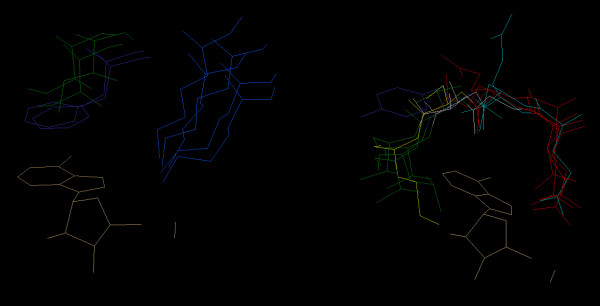
**Superimposition of residues associated to the ADP4 pattern**. Superimposition of residues (Wireframe representation and Amino acid/Shapely colour scheme) which have atoms belonging to the pattern. a) [FI] [VLI] [K] part of the pattern ([VLI] is not part of the structural pattern). b) [EQ]-X-[ACVY] [MLV] part of the pattern

The sequences for these structures can be manually aligned with Jalview [22], using these constraints; see Figure 2 where the pattern is highlighted. This reveals the remote similarity between 1iah and the other proteins of the ADP4 pattern. Figure 3 shows the 3D pattern and Figure 4 shows the superimposition of residues whose atoms belong to the pattern.

Taking into account that 1iah structure has a protein kinase-like fold, it is intriguing why our method clustered the 1iah ATP binding site together with structures from the ATP-grasp fold rather than with other protein kinases clustered in AxP1 (Table 2). ATP molecules bind in the cleft between the N- and C-terminal lobs in all ADP4 and AxP1 proteins. Detailed structural analysis [23] revealed that 1iah has a chimeric structure where the N-terminal domain is very similar to domains of classical protein kinases whilst the C-terminal one is similar to domains of the ATP-grasp fold. In that respect the 1iha structure makes a link between protein kinase and the ATP-grasp folds, which explains why its ATP-binding site was clustered together with those belonging to the ATP-grasp fold. Interestingly, the remote similarity between classical protein kinase folds and the ATP-grasp fold had been noted previously and explained using the concept of either convergent [24] or divergent [25] evolution. We thus believe that the detected pattern is meaningful.

### Towards protein classification, function annotation & putative site discovery

Possible applications of 3D patterns and motifs are protein classification, functional annotation and the discovery of putative binding sites. In order to evaluate the classification and annotation power of the patterns generated by our method, each of them was used to query all PDB proteins binding purine based ligands. The first targets were all the AMP, ADP and ATP binding proteins. We then added proteins binding two very similar ligands: ANP and ACP. Finally, we looked at quite a different family of ligands with three guanine based ligands: GMP, GDP and GTP. The total number of PDB entries per ligand as well as the search results are shown in Table 4.

**Table 4:** Total number of PDB entries per ligand and matches against the generated 3D patterns

	**AMP**	**ADP**	**ATP**	**ANP**	**ACP**	**GMP**	**GDP**	**GTP**
PDB entries containing ligand	128	406	234	125	29	27	199	77
Hits against the 13 patterns	14.8%	30.5%	35.5%	36.8%	44.8%	0.0%	0.5%	1.3%
PDB50 entries in valid clusters	16.0%	30.8%	23.3%	/	/	/	/	/

During the search procedure those proteins whose similarity was higher than the automatic similarity threshold were classified as positive hits, see Table 4. For each of these proteins, its annotations, i.e. functions, EC numbers, fold classifications and sequence motifs, were compared manually with those of the pattern and/or of the proteins used to define the pattern. If there was an exact match, the protein was set as true positive; otherwise it was set as false positive. Since this validation required human expertise, only the positive hits were analysed.

**Figure 5:**
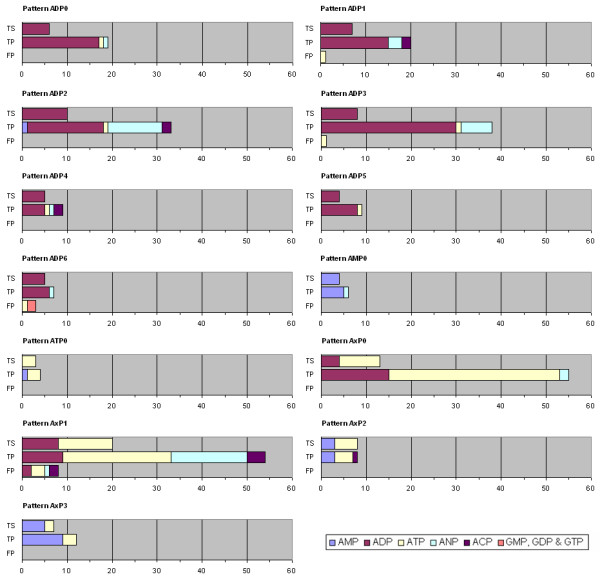
**Composition of Training Sets (TS), True Positives (TP) & False Positives (FP) against all PDB proteins binding either purine based or guanine based ligands**. X-axis gives the number of binding sites which are present in each set. Their type, i.e. AMP, ADP, ATP, ANP, ACP, GMP, GDP or GTP binding, is represented by different colours (see legend).

Figure 5 presents the results for the evaluation of classification power of the 3D patterns. First, the size and composition of the training set (TS) of each pattern is given. Composition is defined according to the type of ligands which are bound by its core proteins. Secondly, the number and composition of true positives (TP) are shown. Finally, false positives (FP), if any, are described.

Approximately 33% of ADP and ATP proteins and 15% of AMP proteins matched the 3D patterns, see Table 4. These rates correspond to the percentage of binding sites in the PDB50 set, which were originally present in valid clusters when patterns were generated, see Table 4. The rate for ATP proteins is, however, higher than expected because their binding sites often match patterns generated from ADP clusters, see Figure 5. Since physicochemical properties of ANP and ACP are very similar to ADP, proteins binding these ligands were very well classified using motifs generated independently from their binding sites. The high percentage of hits against the patterns for ACP binding sites is not significant because the ACP sample is particularly small (29 entries). As a whole, our 3D patterns matched 31% of proteins with adenine based ligands. Since guanine and adenine molecules are chemically very different, proteins with guanine based ligands were not expected to be matched by our adenine patterns. In fact, only two hits were produced from more than 300 of these proteins. Finally, the rate of false positive is very low (4.5%).

These results were obtained using automatically generated similarity thresholds, which proved quite conservative. By setting these thresholds manually to optimise the number of proteins matched by each pattern, the number of adenine proteins matched was increased to 38% while maintaining a low rate of false positives (6.5%).

Although some patterns such as AMP0, AxP2, ADP6 and ATP0 mainly detect the very small number of proteins which were used to produce them, other patterns such as ADP2, ADP3, AxP0 and AxP1 are able to hit a large number of proteins with a low number of false positives. These 3D patterns, therefore, show good potential for protein annotation.

Although DALI would be a better function predictor on this dataset – 100% success rate -, it relies on a very dense sampling of the known protein structure space. Indeed, the annotation of the 281 protein structures containing adenine based ligands required 114 different structure representatives, which correspond to 40.5% of the number of structures to annotate. Since our 13 3D motifs contain only a combined total of 368 atom positions, there is a 100-fold difference between the amount of data stored to produce those predictions. This supports our claim that our motifs capture atom positions which are key to binding site activity.

Since our method can be applied on any ligand containing some rigid structure, many other ligand families could be studied. 3D motifs could be generated, for example, from haem and chlorophyll groups, monosaccharides (e.g. glucose, mannose, frucose, galactose and NAG) and other common rigid ligands such as PLP, FMN, PCA and MES since they are present in many PDB entries.

## Conclusion

We have presented a new metric which permits the classification of proteins according to the atomic environment of binding sites. The only constraint is that their ligands should contain some rigid structure so that they can be superimposed. By studying proteins binding adenine based ligands, we have demonstrated that the core of the generated clusters are biochemically meaningful and can be used to generate useful 3D motifs. We have shown that these motifs are efficient for protein classification and annotation since their false positive rate is low. In addition, they are quite ligand specific. Since our method was able to rediscover a pattern in the case of ADP4 revealing complex evolutionary links between two classes of proteins, we believe our technique could also be used to detect cases of convergent evolution.

In future work we plan to develop a software tool which would permit fast and efficient parsing of 3D protein structures to detect putative binding sites corresponding to our atom based 3D motifs.

## Method

### Terminology used in this work

Atom – in this work we only consider non-hydrogen atoms. Their coordinates are retrieved from PDB files.

Ligand – molecule interacting with a protein in either a non-covalent or a covalent fashion – prosthetic group. In this work we only consider ligands containing rigid 3D structures such as aromatic rings.

Ligand binding site – subset of protein atoms that are situated within a distance of 5.0 Å from at least one ligand atom. Since proteins often contain several binding sites involving the same type of ligand, only one binding site per PDB entry is considered in order not to introduce any bias during the active site clustering process.

3D pattern – consensus atom positions generated from superimposition of ligand binding sites.

3D motif – 3D pattern associated with a biochemical function. In this work, biochemical function is defined by either a consensus functional annotation or a consensus sequence motif.

### Outline of the methodology

Our method comprises the following steps:

• generation of ligand-specific training sets of binding sites,

• comparison of the binding sites of the training set proteins,

• clustering of these proteins according to the results of their binding site comparison

• generation of patterns representing each of the clusters.

**Figure 6:**
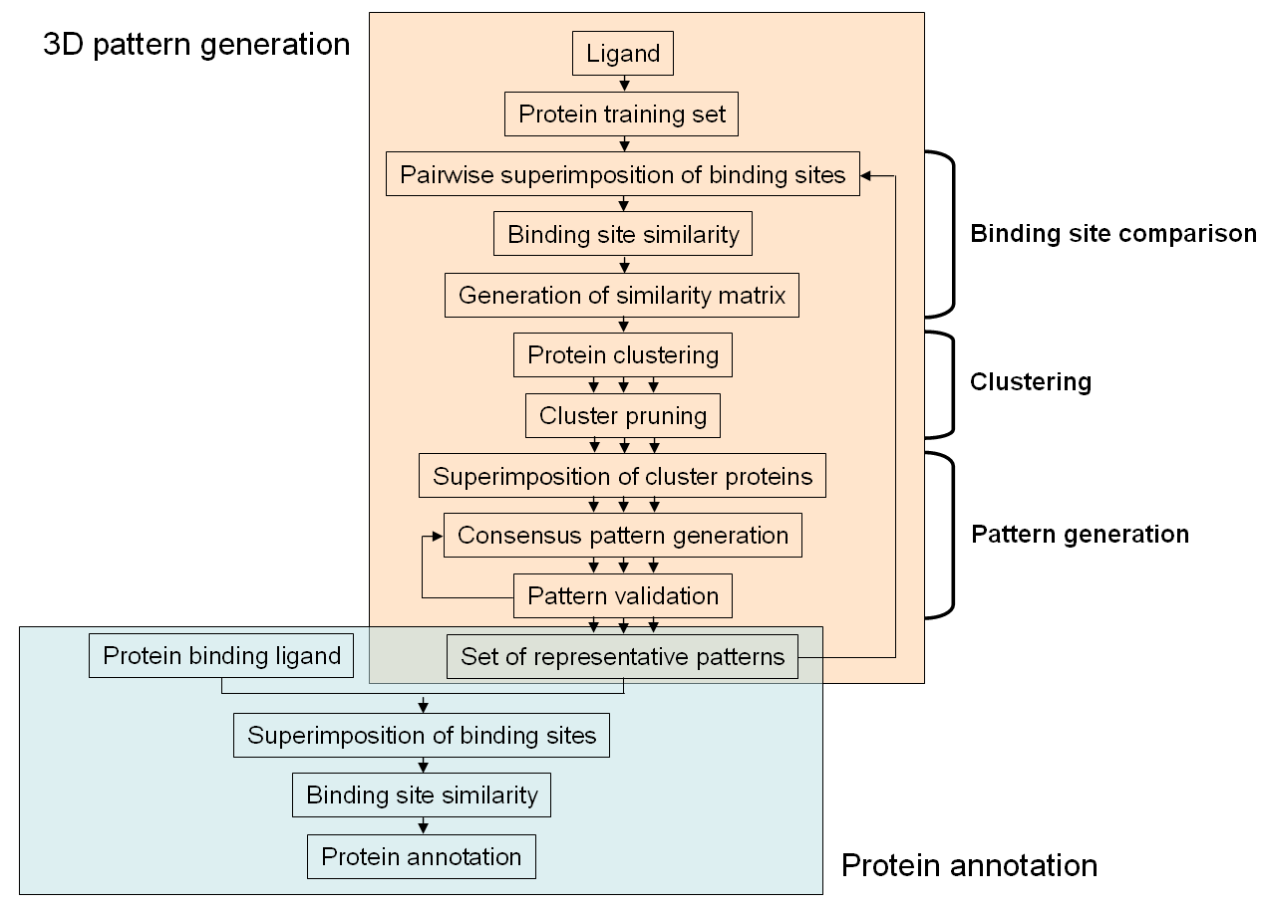
Outline of the methodology used for the generation of 3D patterns and protein annotation.

Figure 6 provides a detailed description of the methodology.

### Protein structure data sets

For the purpose of 3D pattern generation the PDB50%, a set of PDB protein structures trimmed so that no single pair of proteins has sequence identity higher than 50% [15], was used. PDB50% was chosen because it offers a good compromise between providing a sufficient number of PDB entries for each ligand of interest and preventing the dataset to be too biased by close homologs. For each of the main adenine based ligands, namely AMP, ADP and ATP a training set was constructed, representing a set of ligand binding sites extracted from the subset of PDB50% that binds a particular ligand. The sizes of the training sets generated on 6^th ^December 2005 are presented in Table 1.

For the purpose of classification evaluation we used a set of protein structures that bind purine based ligands, namely AMP, ADP, ATP, ANP, ACP, GMP, GDP and GTP, selected from the entire PDB set. The numbers of PDB entries for each of these ligands are presented in Table 4.

### Binding site comparison

Binding sites of the training set were compared to one another in one-to-one comparisons. Binding sites were superimposed by performing rigid transformations between them according to the atom positions of the rigid structure of their ligands. An algorithm developed by Horn [26] was implemented to determine the translation and rotation that will align atoms in one coordinate system to corresponding atoms in another coordinate system, while minimizing the RMSD between the two fitted sets of atoms. Subsequently, for each pairwise comparison those atoms that could not be paired within a given threshold with atoms of the same chemical type (carbon, oxygen, nitrogen or sulphur) were discarded.

Consensus sites were then used to estimate the similarity between the protein binding sites. The similarity of two sites A and B, S_AB_, was calculated using Kulczynski's metric:

SAB=0.5(CABTA+CABTB)
 MathType@MTEF@5@5@+=feaafiart1ev1aaatCvAUfKttLearuWrP9MDH5MBPbIqV92AaeXatLxBI9gBaebbnrfifHhDYfgasaacH8akY=wiFfYdH8Gipec8Eeeu0xXdbba9frFj0=OqFfea0dXdd9vqai=hGuQ8kuc9pgc9s8qqaq=dirpe0xb9q8qiLsFr0=vr0=vr0dc8meaabaqaciaacaGaaeqabaqabeGadaaakeaacqWGtbWudaWgaaWcbaGaemyqaeKaemOqaieabeaakiabg2da9iabicdaWiabc6caUiabiwda1maabmaabaWaaSaaaeaacqWGdbWqdaWgaaWcbaGaemyqaeKaemOqaieabeaaaOqaaiabdsfaunaaBaaaleaacqWGbbqqaeqaaaaakiabgUcaRmaalaaabaGaem4qam0aaSbaaSqaaiabdgeabjabdkeacbqabaaakeaacqWGubavdaWgaaWcbaGaemOqaieabeaaaaaakiaawIcacaGLPaaaaaa@4224@

where C_AB _is the number of atoms within the pairwise consensus ligand binding site and T_A _(resp. T_B_) is the number of atoms in protein A (resp. B) within its ligand binding site.

The above similarity matrix S was then used to cluster the set of proteins.

### Clustering of ligand binding sites

The clustering process is two-fold: first, binding sites are clustered and secondly the generated clusters are pruned to remove outliers. The first stage is achieved by a state of the art general purpose graph-partitioning based algorithm, CLUTO [27,28], which has already been used in a variety of bioinformatics applications [29-33]. Since CLUTO is a partition algorithm, its result depends on the number of partitions (k) and the final clusters may contain outliers. However, these potential limitations do not significantly affect our system. Since the aim of the clustering process is the generation of tight protein clusters which will be subsequently used for 3D pattern generation, not all proteins need to belong to a cluster. Therefore, clusters are post processed so that only cores of the most compact clusters are kept, see next paragraph. Moreover, our method allows clusters to be merged at a later stage of the process. Therefore, the choice of k is not a critical step. In this study k was set at 10% of the number of sites in the training set so that the clusters generated were big enough to generate meaningful consensus patterns while their internal similarity remained high. Cluster quality could be monitored since for each cluster CLUTO provides its average internal and external similarities and their associated standard deviations.

The second stage of the process is a pruning process, which removes outliers from each cluster. Since binding site relationships are expressed by a similarity matrix, outlier detection is achieved by a distance-based technique [34], where outliers are defined as data points whose similarity to the data centroid is lower than a given threshold value. Since the clusters generated by our method are rather small – less than 100 members – outliers may impact significantly on the centroid position. Consequently, our method employs an iterative process, which implicitly calculates the new position of the centroid after an outlier is removed from a cluster.

Within a cluster of n binding sites, we calculate for each member, i, its average similarity, S_i_, with all the other members of the cluster. The binding site with the lowest average similarity is discarded if it is below a given similarity threshold, S_T_.

Si=1n−1∑j=1,j≠inSij
 MathType@MTEF@5@5@+=feaafiart1ev1aaatCvAUfKttLearuWrP9MDH5MBPbIqV92AaeXatLxBI9gBaebbnrfifHhDYfgasaacH8akY=wiFfYdH8Gipec8Eeeu0xXdbba9frFj0=OqFfea0dXdd9vqai=hGuQ8kuc9pgc9s8qqaq=dirpe0xb9q8qiLsFr0=vr0=vr0dc8meaabaqaciaacaGaaeqabaqabeGadaaakeaacqWGtbWudaWgaaWcbaGaemyAaKgabeaakiabg2da9maalaaabaGaeGymaedabaGaemOBa4MaeyOeI0IaeGymaedaamaaqahabaGaem4uam1aaSbaaSqaaiabdMgaPjabdQgaQbqabaaabaGaemOAaOMaeyypa0JaeGymaeJaeiilaWIaemOAaOMaeyiyIKRaemyAaKgabaGaemOBa4ganiabggHiLdaaaa@4515@

Site i is an outlier, if Si=Minj=1n(Sj)
 MathType@MTEF@5@5@+=feaafiart1ev1aaatCvAUfKttLearuWrP9MDH5MBPbIqV92AaeXatLxBI9gBaebbnrfifHhDYfgasaacH8akY=wiFfYdH8Gipec8Eeeu0xXdbba9frFj0=OqFfea0dXdd9vqai=hGuQ8kuc9pgc9s8qqaq=dirpe0xb9q8qiLsFr0=vr0=vr0dc8meaabaqaciaacaGaaeqabaqabeGadaaakeaacqWGtbWudaWgaaWcbaGaemyAaKgabeaakiabg2da9iabd2eanjabdMgaPjabd6gaUnaaDaaaleaacqWGQbGAcqGH9aqpcqaIXaqmaeaacqWGUbGBaaGcdaqadaqaaiabdofatnaaBaaaleaacqWGQbGAaeqaaaGccaGLOaGaayzkaaaaaa@3D8F@ and *S*_*i *_<*S*_*T*_

### Pattern generation

The general idea behind the generation of a 3D pattern representing a given cluster is to superimpose all the binding sites of a given cluster and generate a cluster consensus ligand binding site. Then the 3D pattern is tested against all binding sites of the training set to evaluate if it is representative of the associated cluster. This task is performed by ranking all binding sites according to their similarity with the pattern of interest after superimposition of their ligands. The top hits should consist of the binding sites of the cluster members – true positives. If there is any false positive, the pattern is optimised so that the rank of the first false positive is the lowest. Finally, when a pattern is generated for a given cluster, its similarity to the last true positive is stored as a conservative threshold which will permit the automatic decision if an active site can be annotated with the properties associated with the cluster.

Consensus patterns for sequences and secondary structure elements are usually generated by a hierarchical process of pairwise comparisons [17]. However, since atom positions consist of continuous values, pairwise comparisons are not transitive. Therefore, a 3D pattern generation can only be generated after performing all pairwise superimpositions of binding sites which are present in a given cluster. After each comparison, only consensus atoms are kept within the pair of binding sites. This is performed using the technique described in the 'binding site comparison' section. At the end of the process, each binding site is only composed of atoms whose positions are similar within the whole cluster. The 3D pattern is then generated by averaging atom positions from all the binding sites.

The process of generation of a pattern P is described below in pseudo code where n is the number of binding sites, BS, in a given cluster:

   For i = 1 to n-1

   For j = i+1 to n

      Superimpose(BSi, BSj)

      Generate consensus binding sites Ci & Cj

      BSi = Ci

      BSj = Cj

   endFor

endFor

For i = 1 to n

   P = BSi/n

endFor

A more detailed description of the 3D pattern generation technique is given in [13].

We define a "good quality pattern" as a pattern containing the minimum number of atoms which allows the discrimination of the binding sites of the cluster from all the binding sites of the training set. While cluster consensus patterns contain the minimum number of atoms whose positions are shared among the binding sites of the cluster, they may not be fully discriminative, i.e. there may be at least one false positive ranking higher than the last true positive. In such cases, new less compact patterns are produced to achieve better binding site discrimination. A new cluster pattern is generated using the process previously described without including the binding site which is the furthest away from the cluster centroid. Although this binding site was not involved in the pattern generation, the discrimination power of the pattern is still evaluated for all active site in the cluster. This process is iterated until a fully representative pattern is generated or only two active sites are left for pattern generation. In the latter case, the most representative pattern is retained. Although this process does not ensure that selected patterns provide the best representation of a cluster, in practice they prove extremely good for binding site discrimination (see 'Results and discussion' section).

Once patterns are generated for all clusters, they are superimposed in a pairwise fashion to calculate their similarity. The clusters associated with highly similar patterns are merged and new 3D patterns are generated for each of the new clusters.

## Authors' contributions

DRG and JCN designed the clustering and pattern generation methodology. JCN implemented the methodology and processed data sets. PH and JCN performed data analysis. PH produced biological interpretations. All authors contributed to draft the manuscript. All authors read and approved the final manuscript.

## Supplementary Material

Additional file 1 Cluster composition. File providing the list of PDB proteins defining each cluster. Click here for file

Additional file 2 ADP0. File containing the coordinates and types of atoms belonging to the 3D motif named ADP0. It also includes atoms belonging to the ligand. Click here for file

Additional file 3 ADP1. File containing the coordinates and types of atoms belonging to the 3D motif named ADP1. It also includes atoms belonging to the ligand. Click here for file

Additional file 4 ADP2. File containing the coordinates and types of atoms belonging to the 3D motif named ADP2. It also includes atoms belonging to the ligand. Click here for file

Additional file 5 ADP3. File containing the coordinates and types of atoms belonging to the 3D motif named ADP3. It also includes atoms belonging to the ligand. Click here for file

Additional file 6 ADP4. File containing the coordinates and types of atoms belonging to the 3D motif named ADP4. It also includes atoms belonging to the ligand. Click here for file

Additional file 7 ADP5. File containing the coordinates and types of atoms belonging to the 3D motif named ADP5. It also includes atoms belonging to the ligand. Click here for file

Additional file 8 ADP6. File containing the coordinates and types of atoms belonging to the 3D motif named ADP6. It also includes atoms belonging to the ligand. Click here for file

Additional file 9 AMP0. File containing the coordinates and types of atoms belonging to the 3D motif named AMP0. It also includes atoms belonging to the ligand. Click here for file

Additional file 10 ATP0. File containing the coordinates and types of atoms belonging to the 3D motif named ATP0. It also includes atoms belonging to the ligand. Click here for file

Additional file 11 AxP0. File containing the coordinates and types of atoms belonging to the 3D motif named AxP0. It also includes atoms belonging to the ligand. Click here for file

Additional file 12 AxP1. File containing the coordinates and types of atoms belonging to the 3D motif named AxP1. It also includes atoms belonging to the ligand. Click here for file

Additional file 13 AxP2. File containing the coordinates and types of atoms belonging to the 3D motif named AxP2. It also includes atoms belonging to the ligand. Click here for file

Additional file 14 AxP3. File containing the coordinates and types of atoms belonging to the 3D motif named AxP3. It also includes atoms belonging to the ligand. Click here for file

## References

[B1] Murzin AG, Brenner SE, Hubbard T, Chothia C (1995). SCOP: a structural classification of proteins database for the investigation of sequences and structures. J Mol Biol.

[B2] Holm L, Sander C (1996). Mapping the protein universe. Science.

[B3] Pearl FMG, Lee D, Bray JE, Sillitoe I, Todd AE, Harrison AP, Thornton JM, Orengo CA (2000). Assigning genomic sequences to CATH. Nucleic Acids Research.

[B4] Golovin A, Oldfield TJ, Tate JG, Velankar S, Barton GJ, Boutselakis H, Dimitropoulos D, Fillon J, Hussain A, Ionides JMC, John M, Keller PA, Krissinel E, McNeil P, Naim A, Newman R, Pajon A, Pineda J, Rachedi A, Copeland J, Sitnov A, Sobhany S, Suarez-Uruena A, Swaminathan J, Tagari M, Tromm S, Vranken W, Henrick K (2004). E-MSD: an integrated data resource for bioinformatics. Nucleic Acids Research.

[B5] Stark A, Sunyaev S, Russell RB (2003). A model for statistical significance of local similarities in structure. J Mol Biol.

[B6] Porter CT, Bartlett GJ, Thornton JM (2004). The Catalytic Site Atlas: a resource of catalytic sites and residues identified in enzymes using structural data. Nucl Acids Res.

[B7] Madsen D, Kleywegt GJ (2002). Interactive motif and fold recognition in protein structures. J Appl Cryst.

[B8] Liang MP, Banatao DR, Klein TE, Brutlag DL, Altman RB (2003). WebFEATURE: an interactive web tool for identifying and visualizing functional sites on macromolecular structures. Nucleic Acids Res.

[B9] Jambon M, Imberty A, Deléage G, Geourjon G (2003). A new bioinformatic approach to detect common 3D sites in protein structures. Proteins.

[B10] Sigrist CJA, Cerutti L, Hulo N, Gattiker A, Falquet L, Pagni M, Bairoch A, Bucher P (2002). PROSITE: a documented database using patterns and profiles as motif descriptors. Brief Bioinform.

[B11] Mulder NJ, Apweiler R, Attwood TK, Bairoch A, Bateman A, Binns D, Bradley P, Bork P, Bucher P, Cerutti L, Copley R, Courcelle E, Das U, Durbin R, Fleischmann W, Gough J, Haft D, Harte N, Hulo N, Kahn D, Kanapin A, Krestyaninova M, Lonsdale D, Lopez R, Letunic I, Madera M, Maslen J, McDowall J, Mitchell A, Nikolskaya AN, Orchard S, Pagni M, Ponting CP, Quevillon E, Selengut J, Sigrist CJ, Silventoinen V, Studholme DJ, Vaughan R, Wu CH (2005). InterPro, progress and status in 2005. Nucleic Acids Res.

[B12] Livingstone CD, Barton GJ (1993). Protein sequence alignments: a strategy for the hierarchical analysis of residue conservation. Comput Appl Biosci.

[B13] Nebel JC (2006). Generation of 3D templates of active sites of proteins with rigid prosthetic groups. Bioinformatics.

[B14] Laskowski RA, Chistyakov VV, Thornton JM (2005). PDBsum more: new summaries and analyses of the known 3D structures of proteins and nucleic acids. Nucleic Acids Res.

[B15] Berman HM, Westbrook J, Feng Z, Gilliland G, Bhat TN, Weissig H, Shindyalov IN, Bourne PE (2000). The Protein Data Bank. Nucleic Acids Research.

[B16] Gibrat JF, Madej T, Bryant SH (1996). Surprising similarities in structure comparison. Curr Opin Struct Biol.

[B17] Higgins D, Thompson J, Gibson T, Thompson JD, Higgins DG, Gibson TJ (1994). CLUSTALW: improving the sensitivity of progressive multiple sequence alignment through sequence weighting, position-specific gap penalties and weight matrix choice. Nucleic Acids Res.

[B18] Edgar RC (2004). MUSCLE: multiple sequence alignment with high accuracy and high throughput. Nucleic Acids Research.

[B19] Notredame C, Higgins D, Heringa J (2000). T-Coffee: A novel method for multiple sequence alignments. Journal of Molecular Biology.

[B20] Krissinel E, Henrick K (2004). Secondary-structure matching (SSM), a new tool for fast protein structure alignment in three dimensions. Acta Cryst.

[B21] Guda C, Lu S, Sheeff ED, Bourne PE, Shindyalov IN (2004). CE-MC: A multiple protein structure alignment server. Nucleic Acids Res.

[B22] Clamp M, Cuff J, Searle SM, Barton GJ (2004). The Jalview Java Alignment Editor. Bioinformatics.

[B23] Yamaguchi H, Matsushita M, Naim AC, Kuriyan J (2001). Crystal Structure of the Atypical Protein Kinase Domain of a TRP Channel with Phosphotransferase Activity. Molecular Cell.

[B24] Denossiuk KA, Lehtonen JV, Korpela T, Johnson MS (1998). Two unrelated families of ATP-dependent enzymes share extensive structural similarities about their cofactor binding sites. Protein Science.

[B25] Grishin NV (1999). Phosphatidylinositol phosphate kinese: a link between Protein Kinase and Glutathione Synthase folds. J Mol Biol.

[B26] Horn BKP (1987). Closed-form solution of absolute orientation using unit quaternions. J Optical Soc Am.

[B27] Karypis G (2002). CLUTO a clustering toolkit. Technical Report 02-017.

[B28] Crow JA, Retzel EF (2003). wCLUTO: A Web-Enabled Clustering Toolkit. Plant Physiol.

[B29] Tang C, Zhang A, Pei J (2003). Mining Phenotypes and Informative Genes from Gene Expression Data. Proceedings of SIGKDD'03: August 24–27 2003.

[B30] Nakken S (2004). Finding Functionally Related Genes by Local and Global Analysis of MEDLINE Abstracts. Proceedings of Search and Discovery in Bioinformatics Workshop: July 29th 2004, Sheffield.

[B31] Glazko GV, Mushegian AR (2004). Detection of evolutionarily stable fragments of cellular pathways by hierarchical clustering of phyletic patterns. Genome Biol.

[B32] Balasubramaniyan R, Hüllermeier E, Weskamp N, Kämper J (2005). Clustering of gene expression data using a local shape-based similarity measure. Bioinformatics.

[B33] Ucar D, Parthasarathy S, Asur S, Wang C (2005). Effective Pre-processing Strategies for Functional Clustering of a Protein-Protein Interactions Network. Proceedings of the IEEE 5th Symposium on Bioinformatics & Bioengineering (BIBE05): October 2005.

[B34] Knorr EM, Ng RT, Tucakov V (2000). Distance-Based Outliers: Algorithms and Applications. VLDB journals: Very Large Data Bases.

[B35] Webb EC, NC-IUBMB (1992). Enzyme Nomenclature 1992.

